# Impact of exercise intervention on depression, anxiety, sleep and quality of life in patients with cognitive impairment: a systematic review and network meta-analysis

**DOI:** 10.3389/fpsyt.2025.1666157

**Published:** 2025-11-17

**Authors:** Guangyao Sun, Xingyi Ding, Hongtao Ma, Zhong Zheng

**Affiliations:** 1 College of Education, Beijing Sport University, Beijing, China; 2 Department of Physical Education, Peking University, Beijing, China

**Keywords:** cognitive impairment, exercise, depression, anxiety, sleep, quality of life, network meta-analysis

## Abstract

**Background:**

Exercise, as a non-pharmacological intervention, demonstrates considerable potential for improving depression, anxiety, sleep, and quality of life (QoL) in patients with cognitive impairment. However, the optimal exercise modality remains unclear. This study aimed to evaluate and rank the efficacy of exercise types on these outcomes in patients with cognitive impairment.

**Methods:**

A systematic search of PubMed, Web of Science, Embase, and Cochrane Library was conducted to identify studies published between September 2014 and September 2024. Included studies were randomized controlled trials assessing the impact of exercise on depression, anxiety, sleep, and QoL in cognitively impaired individuals. Depression served as the primary outcome, with anxiety, sleep, and QoL as secondary outcomes. All statistical analyses, including pairwise and network meta-analyses, were performed using R version 4.4.1.

**Results:**

Forty studies involving 2,937 participants were included. Exergaming demonstrated superior effectiveness in reducing depression (SMD = −12.52, 95% CrI: −20.6 to −4.53) and anxiety (SMD = −12.49, 95% CrI: −31.27 to 5.98). Multicomponent exercise (ME) significantly reduced depression (SMD = −8.01, 95% CrI: −11.15 to −3.59), while mind-body exercise (MBE) improved quality of life (SMD = 12.61, 95% CrI: 0.73 to 32.77).

**Conclusion:**

Exergaming proved most effective for reducing depression and anxiety in individuals with cognitive impairment. Multicomponent exercise showed substantial benefits for mood regulation, while mind-body exercise was particularly effective for enhancing QoL.

**Systematic review registration:**

https://www.crd.york.ac.uk/PROSPERO/view/CRD42024607193, identifier CRD42024607193.

## 1 Background

Cognitive impairment affects a growing global population. The World Health Organization reports that approximately 50 million individuals are affected, representing 5% to 8% of the global population (1), with prevalence increasing annually. Cognitive impairment ranges from mild to severe and serves as both a risk factor for dementia and Alzheimer’s disease (AD) and a determinant of daily functioning and quality of life (QoL) (2, 3). Findings from large-scale longitudinal studies indicate that depression, anxiety, and sleep disturbances are closely associated with an elevated risk of cognitive decline (4, 5). Early detection and intervention targeting these psychiatric symptoms are therefore crucial.

Psychiatric symptoms and sleep disorders frequently occur in individuals with cognitive impairment and significantly impact QoL (6, 7). Previous research demonstrates strong positive correlations between the severity of depression, anxiety, sleep disorders, and the extent of cognitive decline (8, 9). These symptoms exacerbate deficits in memory, executive function, attention, language, and visuospatial abilities. Depression, anxiety, sleep quality, and QoL represent both contributing factors to and core manifestations of cognitive dysfunction.

As a non-pharmacological approach, exercise holds considerable potential in slowing or mitigating cognitive deterioration. Evidence from animal models and clinical studies supports exercise benefits on cognitive function in patients with cognitive impairment (10–13). Exercise improves self-care abilities, enhances social interaction, alleviates psychiatric symptoms, and promotes cognitive performance (14, 15). However, the psychosocial and cognitive mechanisms underlying the exercise-cognition relationship remain unclear. The exercise-cognition model suggests that physical activity alleviates psychological symptoms, reduces sleep disorders, and improves QoL in patients with cognitive impairment (16–18). However, existing studies vary considerably in participant characteristics, exercise modalities, intensities, and intervention durations, resulting in heterogeneous findings. Depression, anxiety, sleep quality, and QoL likely mediate the exercise-cognition relationship through independent and interactive pathways. No studies have comprehensively examined these interrelationships simultaneously. Addressing this gap is essential for optimizing exercise-based strategies in this population.

Network meta-analysis (NMA) enables comparison of multiple interventions by synthesizing direct and indirect evidence within a single framework, allowing intervention ranking based on relative efficacy for clinical decision-making 15. We conducted a NMA using randomized controlled trial (RCT) data to compare different exercise interventions and identify the most effective modalities for improving depression, anxiety, sleep quality, and QoL in cognitively impaired individuals.

## 2 Methods

This systematic review was registered in PROSPERO (CRD42024607193), with the protocol published in a peer-reviewed journal. The study followed the PRISMA-NMA guidelines (19).

### 2.1 Search strategy

A comprehensive search of Web of Science, PubMed, Embase, and the Cochrane Library was performed from inception through September 2025. The search strategy was initially designed for PubMed and later tailored to other databases to ensure comprehensiveness and consistency (**Supplementary File S1**).

### 2.2 Eligibility criteria

Included studies met these criteria: RCT design; participants with confirmed cognitive impairment (MMSE <27 or MoCA <26); intervention involving any form of exercise training; control group receiving usual care, health education, or alternative exercise; and reporting at least one outcome of interest (depression, anxiety, sleep, or QoL). Only English-language publications were included.

Studies involving cognitive impairment caused by conditions like epilepsy, multiple sclerosis, diabetes, or psychiatric illnesses (e.g., schizophrenia) were excluded, as these conditions involve additional pathological factors that may confound exercise effects. When multiple publications from the same study reported overlapping outcomes, only the most recent publication was included.

### 2.3 Literature screening

Duplicate records were removed using EndNote X9. Titles, abstracts, and full-text articles were independently reviewed by two researchers to determine study eligibility. Any disagreements were settled through consensus or involvement of a third expert.

### 2.4 Data extraction

Two investigators independently extracted data using a standardized Excel form. Extracted information included first author, publication year, country, participant age, sample size, symptom profiles, diagnostic criteria, exercise protocols (modality, frequency, duration), control interventions, and outcome measures. Any disagreements were settled through consensus or involvement of a third expert.

### 2.5 Risk-of-bias assessment

Two reviewers (SGY and DXY) independently assessed the risk of bias using the Cochrane Risk of Bias Tool version 2.0 (RoB 2.0) (20). Five domains were evaluated: randomization process, deviations from intended interventions, missing outcome data, outcome measurement, and selective reporting. Corresponding authors were contacted for missing data. Studies with unobtainable valid data were excluded. Studies were rated as having low risk, some concerns, or high risk of bias, and any discrepancies were addressed through discussion.

### 2.6 GRADE classification of quality of evidence

Two reviewers (SGY and DXY) independently evaluated evidence quality using the Confidence in Network Meta-Analysis (CINeMA) framework. CINeMA, derived from the Grading of Recommendations Assessment, Development and Evaluation (GRADE) approach, specifically assesses confidence in network meta-analysis estimates. Results were stratified by four outcome domains: sleep, QoL, anxiety, and depression. Evidence quality was classified as high, moderate, low, or very low, with disagreements resolved through discussion.

### 2.7 Statistical analysis

A Bayesian NMA was performed in R (version 4.4.1) to assess the comparative efficacy of exercise interventions on depression, anxiety, sleep, and QoL in cognitively impaired individuals. Unlike traditional pairwise meta-analysis, this approach integrates direct and indirect evidence. The gemtc package provided the Bayesian framework within the rjags environment, with coda for convergence assessment and R2WinBUGS/rjags serving as computational engines. Standardized mean differences (SMDs) with corresponding 95% credible intervals (CrIs) were calculated to estimate effect sizes. Network plots were constructed based on intervention relationships, with nodes representing different interventions and edge thickness reflecting the number of direct comparison studies. Network connectivity was assessed through consistency assumption testing. Inconsistencies between direct and indirect estimates were assessed using node-splitting analysis, with a P-value above 0.05 suggesting no significant inconsistency. A random-effects model was applied with between-study heterogeneity assumed to follow a uniform prior distribution (τ∼U(0,2)). Effect size priors were set as non-informative normal distributions (N(0,10^4^)). Parameter estimation was performed using JAGS with four independent Markov chains, each running 50,000 iterations with the first 20,000 as burn-in to eliminate initial value effects.

Interventions were ranked using SUCRA values, where a larger surface area indicated a greater likelihood of being the most effective option. Publication bias was assessed using comparison-adjusted funnel plots.

## 3 Results

### 3.1 Search results

**Figure 1:**
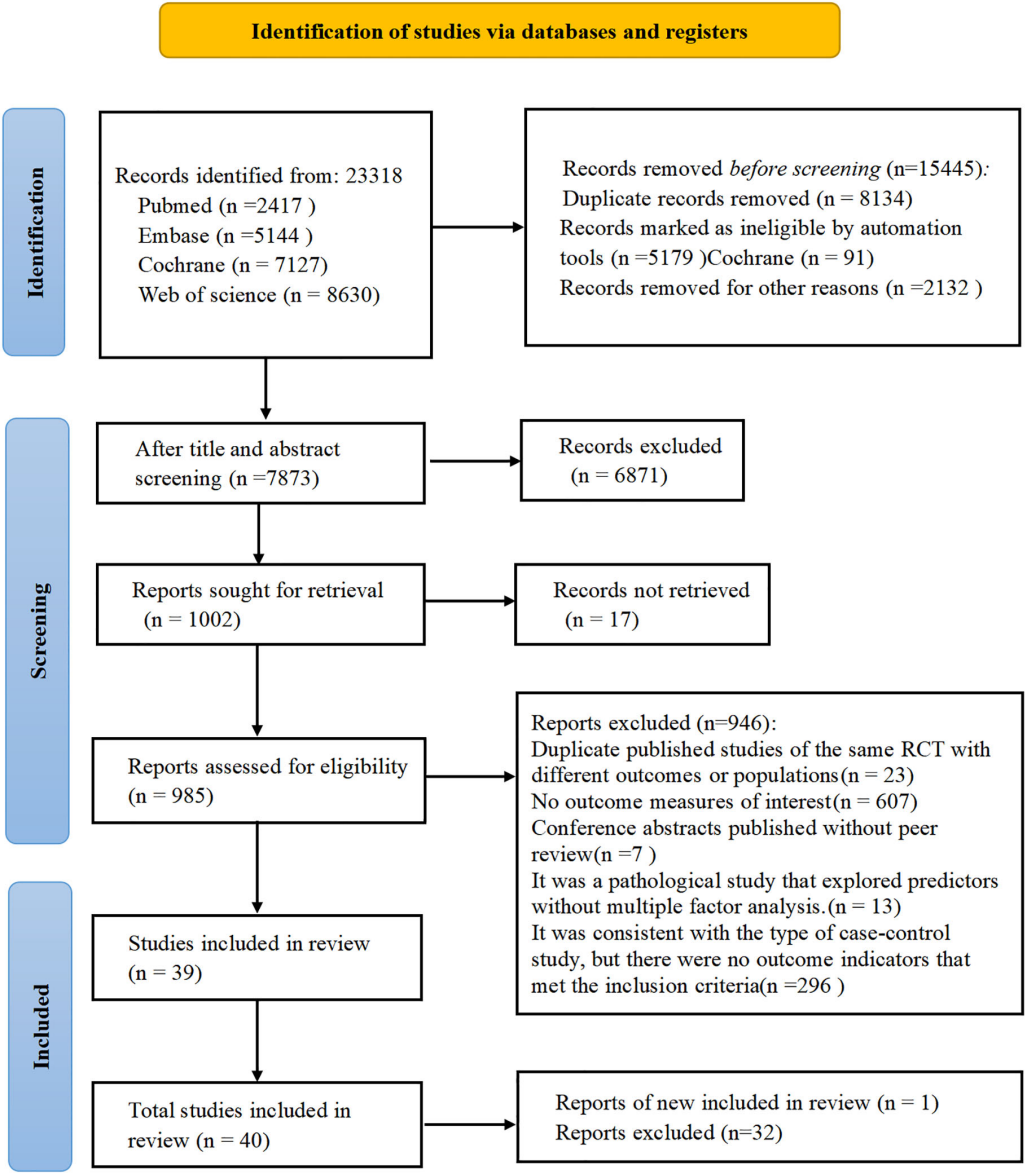
Diagram depicting the process of literature identification and selection.

A total of 23,351 records were retrieved from PubMed, EMBASE, Web of Science, and the Cochrane Library. After removing duplicates and screening for eligibility, 40 studies (11, 21–59) met the inclusion criteria and demonstrated adequate methodological quality (**Figure 1**).

### 3.2 Characteristics of the included studies

The 40 studies involved 2937 participants aged 60 to 90 years. Thirty-six studies focused on depression, 11 studies examined anxiety outcomes, 10 assessed sleep-related effects, and 9 reported QoL outcomes. Participants across the studies were diagnosed with mild cognitive impairment (MCI), dementia, Parkinson’s disease (PD), and AD (**Supplementary Table S1**). A total of 10 types of exercise were included (**Table 1**).

**Table 1:** Exercise type terminology table.

Exercise (Abbreviation)	Exercise (Full Name)	Definition
AE	Aerobic Exercise	Physical activity that involves sustained, rhythmic movement of large muscle groups, such as running, swimming, and cycling, with the primary purpose of improving cardiopulmonary fitness and endurance.
ME	Multicomponent Exercise	A comprehensive training program that combines multiple types of exercise, typically including aerobic exercise, resistance training, balance, and flexibility exercises.
RE	Resistance Exercise	Exercises designed to increase muscle strength and endurance by working against external resistance, such as using dumbbells, resistance bands, or body weight training.
MBE	Mind-body Exercise	This refers to a combination of physical movement, mental focus, and breath control, designed to improve physical and mental health, such as Yoga, Tai Chi, and Pilates.
SE	Strength Exercise	Exercises focused on increasing a muscle’s maximum force output are usually a form of resistance training, but they tend to focus on heavy weights and low repetitions.
Dan	Dance	An art and sport form that uses body movement as its primary means of expression, often accompanied by music, and can improve cardiovascular fitness, coordination, and mood.
SS	Sport Stacking	This is an individual or team competitive sport where specific cups are stacked into a pyramid shape and then retrieved as quickly as possible. It significantly improves hand-eye coordination, reaction speed, and bilateral coordination.
MCGT	Music-Contingent Gait Training	A rehabilitation training method that uses music (usually rhythmic) to cue and guide gait (walking) rhythm to improve walking pattern.
Exergame	-	It is a term combining the words “exercise” and “game”. It refers to electronic games that require physical activity, such as using a motion camera, balance board, or dance mat. It combines physical exercise with interactive gaming.
SLE	Structured Limb Exercise	A planned, repetitive, task-oriented approach to upper or lower limb exercise, often used in neurological rehabilitation (e.g., post-stroke rehabilitation) to improve motor control, coordination, and function.

### 3.3 Risk of bias

**Figure 2:**
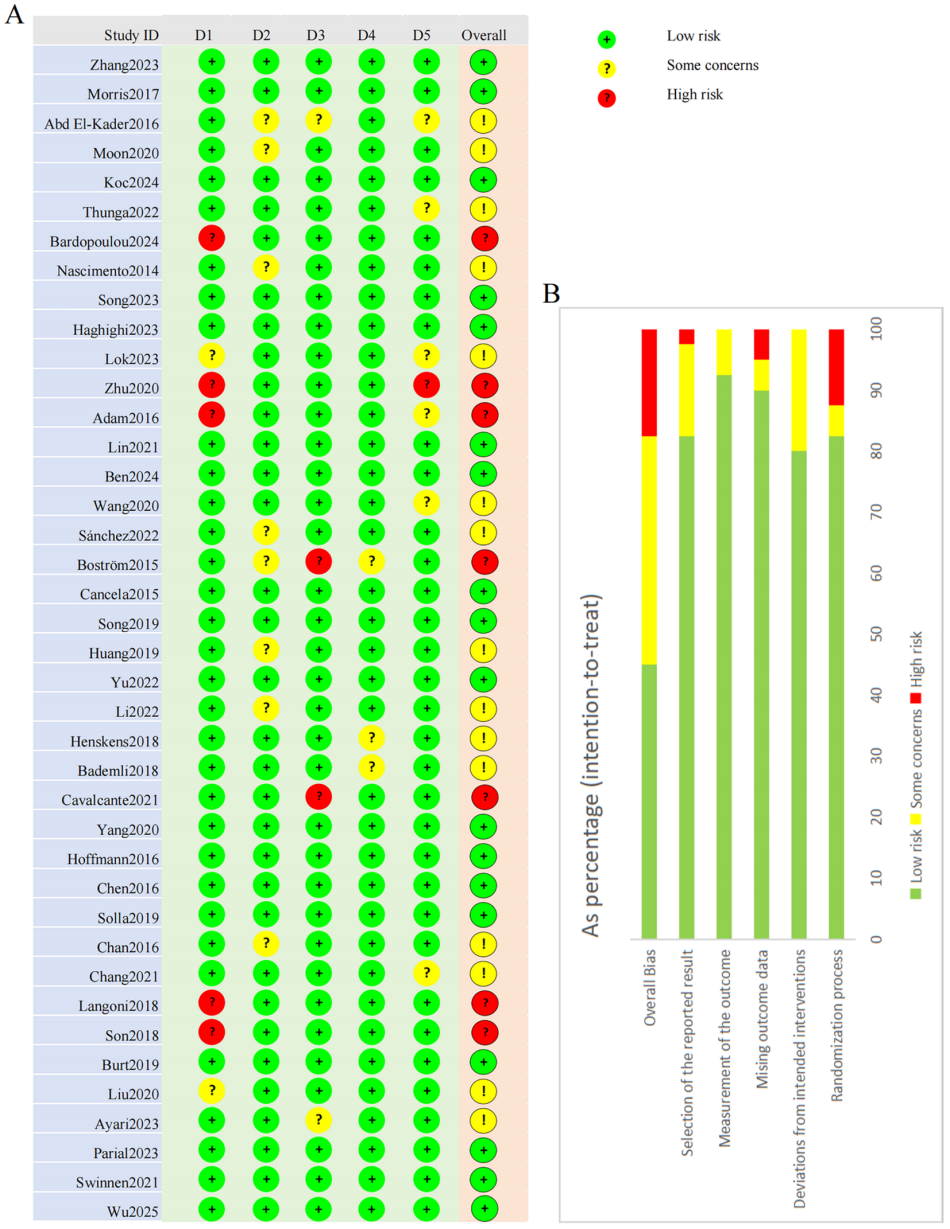
Risk of bias: **(A)** traffic-light plot and **(B)** summary plot.

Risk of bias assessment for all 40 studies is presented in **Figure 2**. Several concerns were identified across multiple domains. Seven studies inadequately described randomization procedures or allocation concealment, potentially affecting baseline comparability and introducing selection bias. Given the inherent features of exercise interventions, participant and personnel blinding was not feasible in any study. Eight studies showed deviations from intended interventions, indicating potential performance bias. Four studies had incomplete outcome data (attrition bias), seven exhibited inadequate outcome measurement procedures (detection bias), and seven had selective reporting concerns, including lack of preregistration or unreported secondary outcomes. Overall, seven studies were rated as high risk of bias, fifteen as having some concerns, and seventeen as low risk.

### 3.4 Outcome measures

#### 3.4.1 Depression

36 studies investigated exercise effects on depression in patients with cognitive impairment. Assessment tools included the Beck Depression Inventory (BDI), Geriatric Depression Scale (GDS), and Hamilton Depression Scale (HAMD). Exercise interventions comprised multicomponent exercise (ME) (8 studies), aerobic exercise (AE) (15 studies), resistance exercise (RE) (3 studies), mind-body exercise (MBE) (5 studies), strength exercise (SE) (3 studies), and dance (5 studies). Individual studies examined sport stacking (SS), musical fitness program, exergaming, music-contingent gait training (MCGT), and RE with instability.

**Figure 3:**
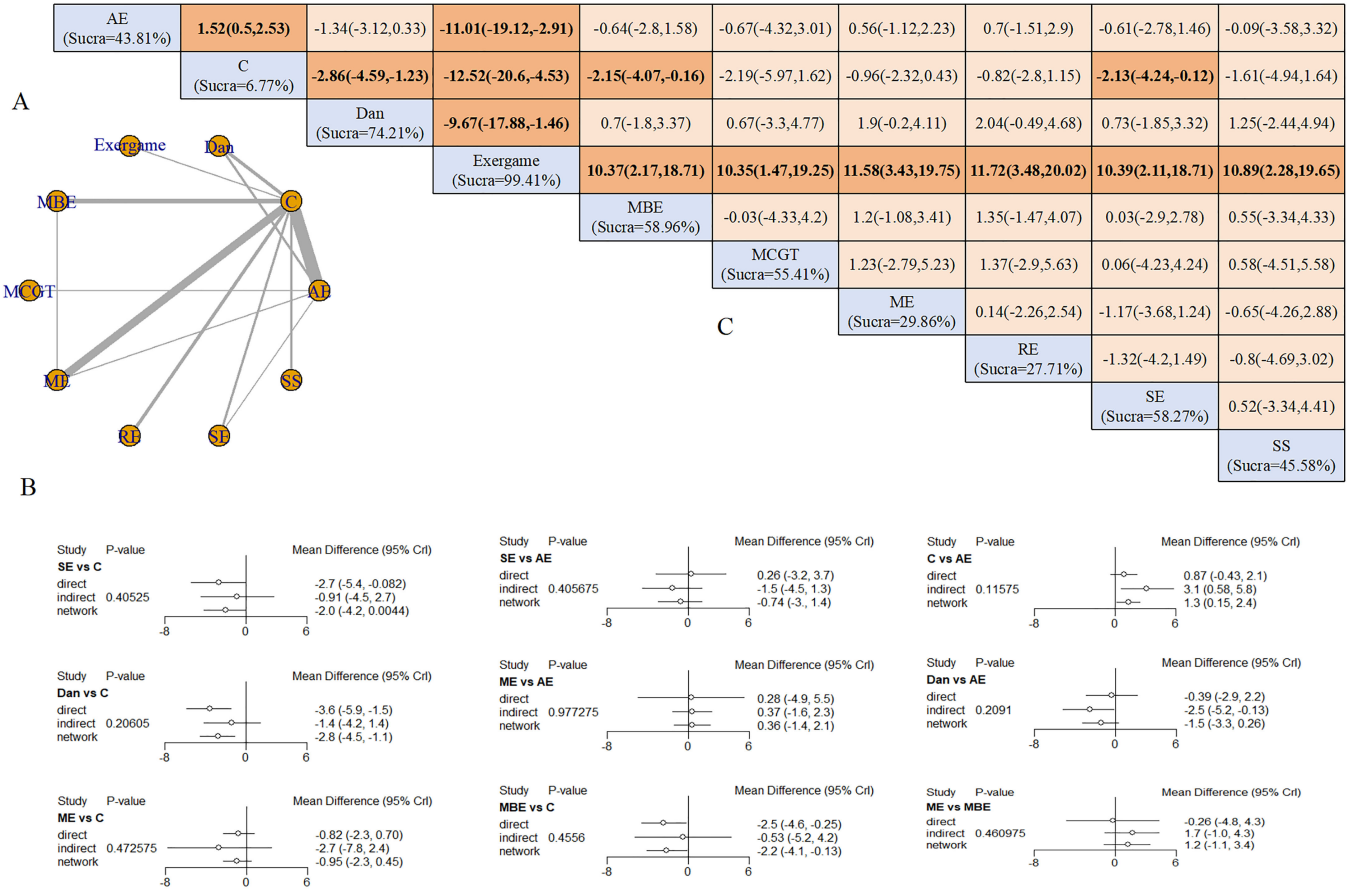
Depression outcomes: **(A)** Network diagram; **(B)** Node-splitting analysis; **(C)** League table and SUCRA ranking.

Nine interventions formed a network centered on the control group, creating four closed loops (**Figure 3A**). Node-splitting analysis revealed no local inconsistency (all P > 0.05) (**Figure 3B**). Five exercise modalities demonstrated significant benefits compared with controls: AE (SMD = −1.52, 95% CrI: −2.53 to −0.50), dance (SMD = −2.86, 95% CrI: −4.59 to −1.23), exergaming (SMD = −12.52, 95% CrI: −20.60 to −4.53), ME (SMD = −2.16, 95% CrI: −4.10 to −0.13), and SE (SMD = −2.13, 95% CrI: −4.24 to −0.12).

Exergaming demonstrated superior efficacy over all other interventions: AE (SMD = −11.01, 95% CrI: −19.12 to −2.91), dance (SMD = −9.67, 95% CrI: −17.88 to −1.46), MBE (SMD = −10.37, 95% CrI: −18.71 to −2.17), MCGT (SMD = −10.35, 95% CrI: −19.25 to −1.47), ME (SMD = −11.58, 95% CrI: −19.75 to −3.43), RE (SMD = −11.72, 95% CrI: −20.02 to −3.48), SE (SMD = −10.39, 95% CrI: −18.71 to −2.11), and SS (SMD = −10.89, 95% CrI: −19.56 to −2.28). SUCRA rankings placed exergaming highest (99.41%), followed by dance (74.21%) and MBE (55.41%) (**Figure 3C**). Sensitivity analysis excluding high-risk studies yielded consistent rankings: exergaming (99.45%), MBE (70.07%), and dance (63.38%).

#### 3.4.2 Anxiety

Eleven studies (458 participants) evaluated exercise effects on anxiety using the Profile of Mood States (POMS), Hospital Anxiety and Depression Scale (HADS), and State-Trait Anxiety Inventory (STAI). Exercise types included ME (2 studies), AE (3 studies), MBE (3 studies), dance (2 studies), MCGT (1 study), RE (1 study), and exergaming (1 study).

**Figure 4:**
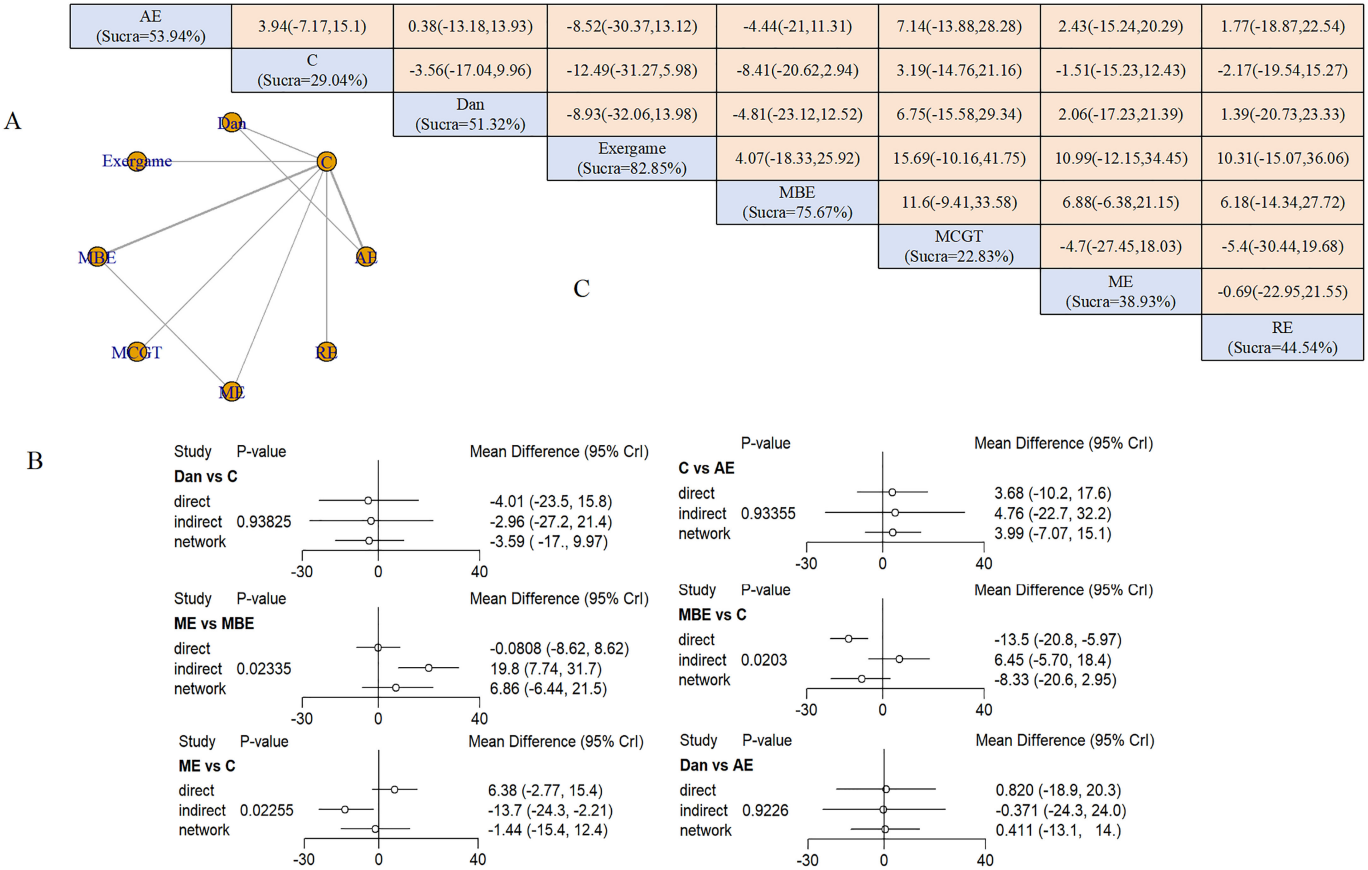
Anxiety outcomes: **(A)** Network diagram; **(B)** Node-splitting analysis; **(C)** League table and SUCRA ranking.

Six interventions formed a network with two closed loops (**Figure 4A**). AE and MBE represented the largest sample sizes. Node-splitting analysis detected inconsistencies between ME and MBE (P = 0.02), ME and control (P = 0.02), and MBE and control (P = 0.02), indicating potential heterogeneity in these comparisons (**Figure 4B**). No intervention achieved statistical significance in direct comparisons. SUCRA values ranked exergaming highest (82.85%), followed by MBE (75.67%) and dance (51.32%) (**Figure 4C**).

#### 3.4.3 Sleep

Ten studies (601 participants) assessed sleep quality using the Pittsburgh Sleep Quality Index (PSQI), Medical Outcomes Study Sleep Scale, and Modified Sleep Questionnaire. Interventions comprised ME (4 studies), AE (2 studies), MBE (3 studies), SE (3 studies), dance (1 study), SS (1 study), and structured limb exercise (1 study).

**Figure 5:**
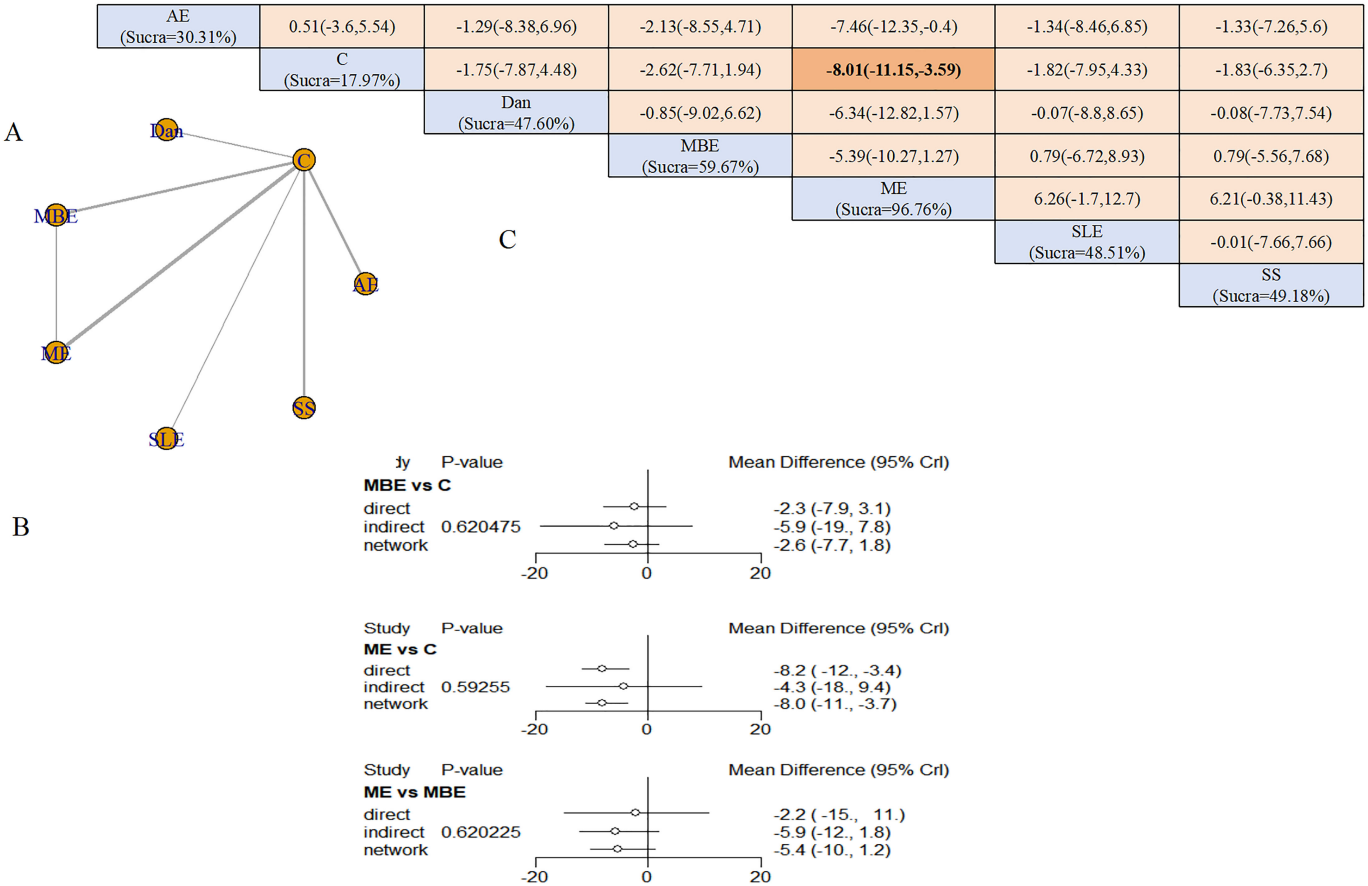
Sleep outcomes: **(A)** Network diagram; **(B)** Node-splitting analysis; **(C)** League table and SUCRA ranking.

A network consisting of six exercise modalities was established around the control group, forming a single closed loop (**Figure 5A**). ME, MBE, and AE represented the interventions with the largest sample sizes among the included studies. Node-splitting analysis confirmed network consistency (all P-values >0.05) (**Figure 5B**).

ME was associated with a substantial improvement in sleep quality relative to the control group (SMD = −8.01, 95% CrI: −11.15 to −3.59). Based on SUCRA analysis, ME demonstrated the greatest probability of being the most effective intervention for improving sleep quality in individuals with cognitive impairment (96.76%), followed by MBE (59.67%) and structured limb exercise (48.51%) (**Figure 5C**).

#### 3.4.4 QoL

Nine studies (685 participants) assessed exercise effects on QoL using the QoL Scale and AD-related QoL instruments. Interventions comprised ME (2 studies), AE (4 studies), MBE (2 studies), RE (1 study), and dance (1 study).

**Figure 6:**
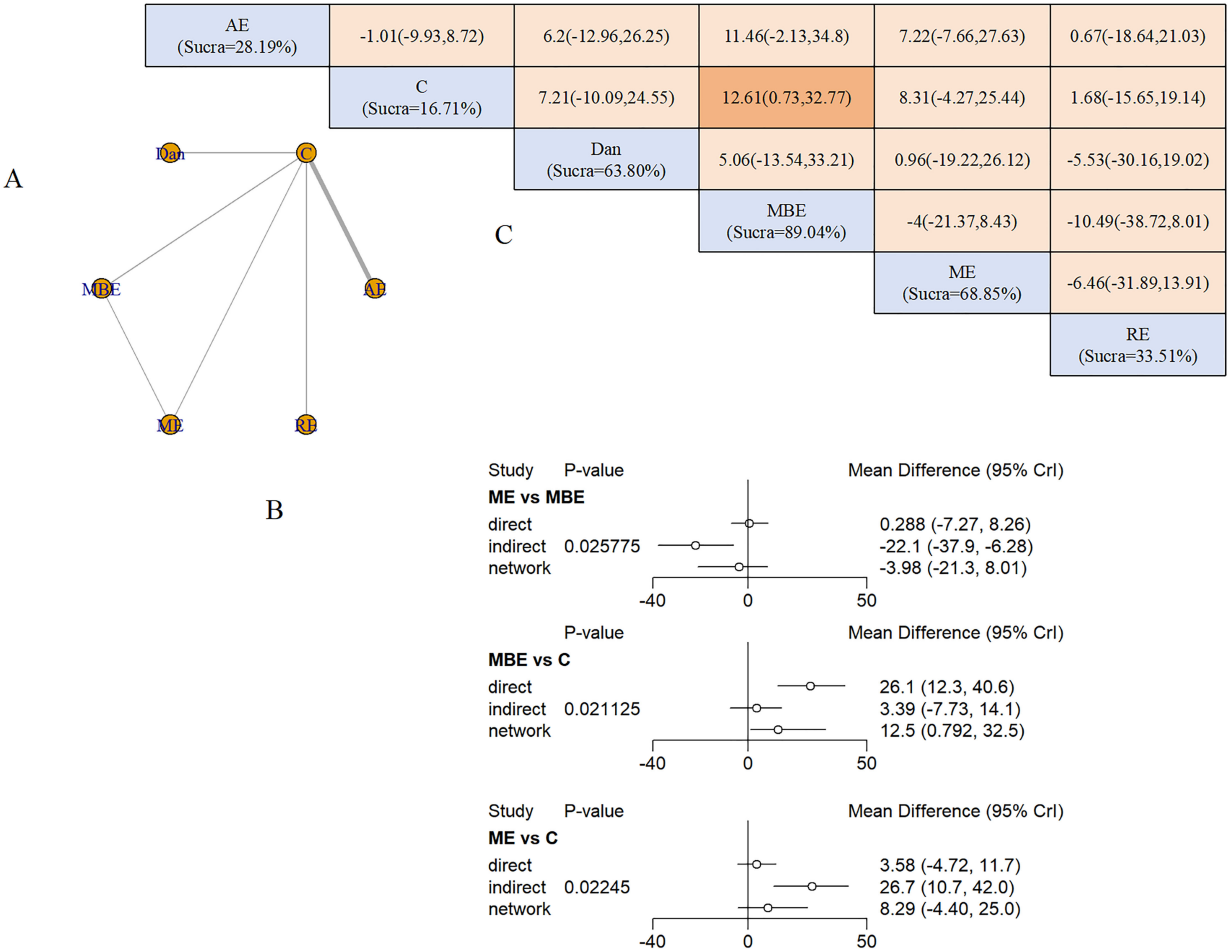
QoL outcomes: **(A)** Network diagram; **(B)** Node-splitting analysis; **(C)** League table and SUCRA ranking.

Four interventions formed a single-loop network (**Figure 6A**), with AE representing the largest sample. Node-splitting analysis detected inconsistencies between ME and MBE (P = 0.02), ME and control (P = 0.02), and MBE and control (P = 0.02) (**Figure 6B**). MBE significantly improved QoL versus controls (SMD = 12.61, 95% CrI: 0.73 to 32.77). SUCRA rankings placed MBE highest (89.04%), followed by ME (68.85%) and dance (63.80%) (**Figure 6C**).

### 3.5 Subgroup analysis of depression

Subgroup analysis examined whether cognitive impairment severity moderated depression outcomes. Studies were stratified into MCI and dementia groups, with model stability confirmed (Deviance Information Criterion (DIC) difference < 5).

**Figure 7:**
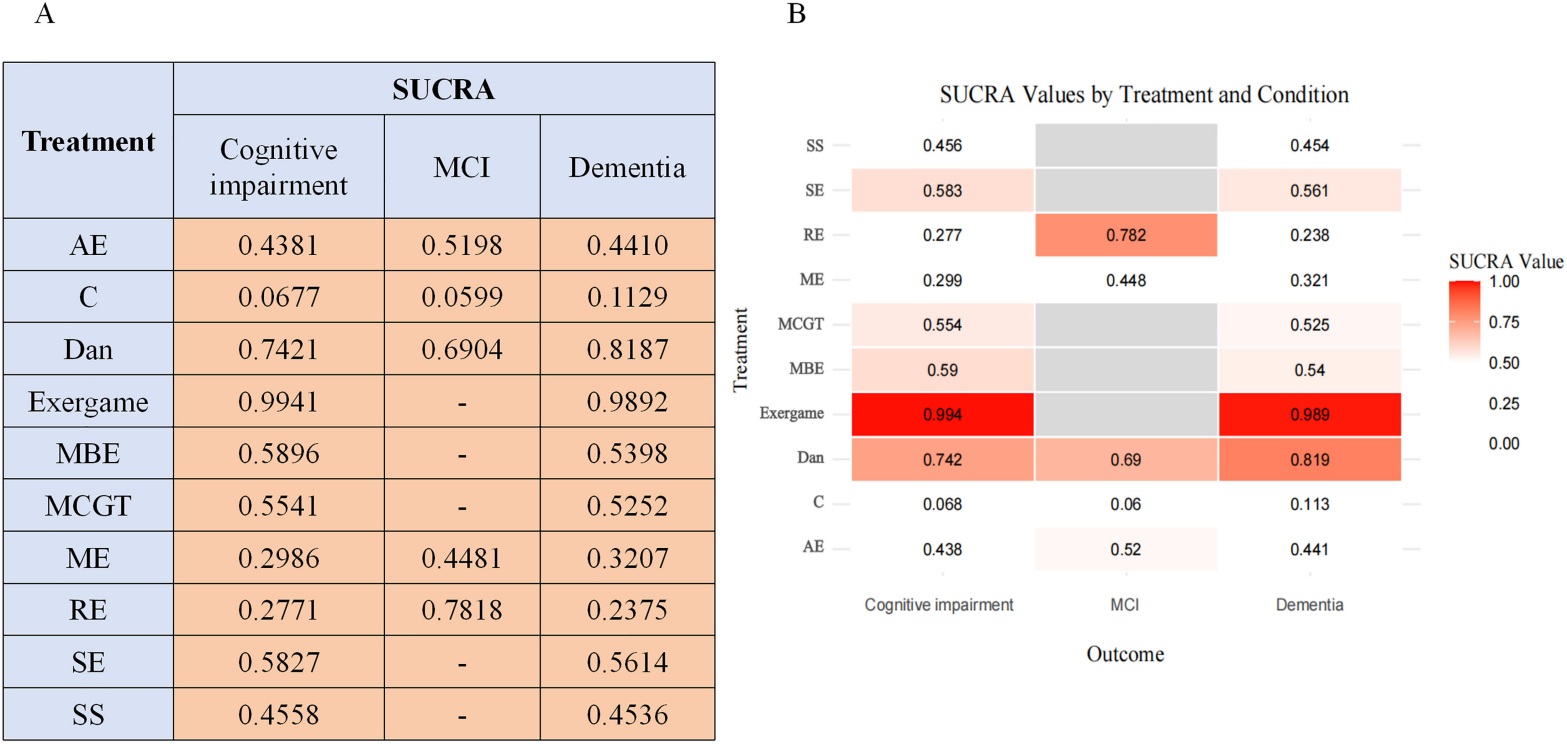
SUCRA probability rankings from subgroup analysis. **(A)** shows the SUCRA values for different treatments across cognitive subgroups (Mild Cognitive Impairment and Dementia). **(B)** shows a heatmap of SUCRA values for each treatment and subgroup. SUCRA values range from 0 to 1, with higher values (the closer to 1.00, the redder the color) indicating a higher ranking and a greater probability of the treatment being superior within that specific subgroup. MCI, Mild Cognitive Impairment.

In dementia, exergaming ranked highest (98.92%), followed by dance (81.87%) and SE (56.14%). In MCI, limited data permitted analysis of four interventions only: RE (78.18%), dance (69.04%), and ME (44.81%)(**Figure 7A**). Overall rankings remained consistent, though dance achieved top-three status in dementia and first position in MCI. While limited exergaming trials in MCI preclude definitive conclusions, evidence supports its efficacy for depression across cognitive impairment severities (**Figure 7B**).

### 3.6 Meta-regression analysis

Bayesian network meta-regression examined how baseline severity, age, and intervention duration moderated treatment effects. Among depression outcomes, intervention duration emerged as the primary driver of heterogeneity, whereas neither age nor baseline depression severity contributed significantly. The effects of aerobic exercise (d.AE.C, beta (1)) and dance (d.AE.Dan, beta (3)) strengthened considerably with longer interventions, evidenced by 95% confidence intervals that did not include zero. These covariates showed no impact on other intervention types. While residual heterogeneity persisted across all models (sd.d > 0), duration alone accounted for some of the observed variation. These findings establish intervention duration as a critical factor influencing depression treatment outcomes, particularly for AE and dance interventions.

Anxiety outcomes presented a different pattern. Neither age, duration, nor baseline anxiety levels accounted for the observed heterogeneity. All models demonstrated substantial residual heterogeneity, with sd.d confidence intervals consistently excluding zero (duration: 0.34–11.74; baseline: 0.17–9.43; age: 0.18–11.66). This unexplained variation suggests the influence of unmeasured factors—potentially intervention intensity, implementation settings, comorbid conditions, or methodological variations—warranting further investigation.

Sleep outcomes similarly showed no association with the three examined covariates. Residual heterogeneity remained evident, with all sd.d values excluding zero (duration: 0.13–9.22; baseline: 0.09–7.09; age: 0.16–8.51), pointing to unaccounted sources of variation.

QoL outcomes followed a comparable pattern. Neither intervention duration, baseline QoL scores, nor age accounted for the heterogeneity. The persistence of significant residual heterogeneity across all models, confirmed by sd.d confidence intervals excluding zero, suggests that unmeasured variables play a substantial role in determining treatment effectiveness.

### 3.7 Sensitivity analysis and publication bias

DIC values for consistency and inconsistency models were: depression (140.35 vs. 141.19), anxiety (39.94 vs. 39.38), sleep (45.32 vs. 46.74), and QoL (33.42 vs. 28.43). All differences were <5, indicating good model consistency and data reliability.

**Figure 8:**
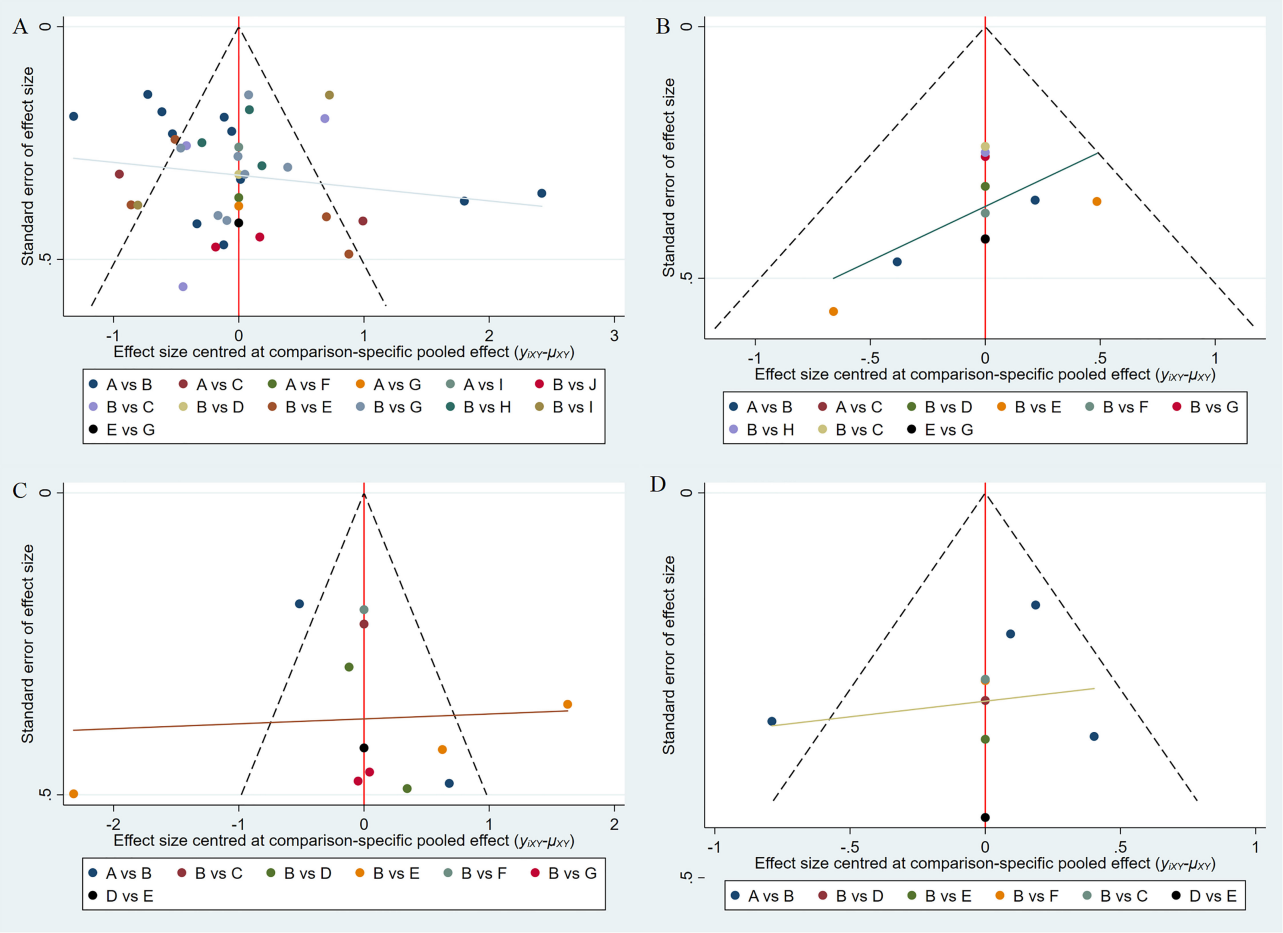
Funnel plots: **(A)** Depression; **(B)** Anxiety; **(C)** Sleep; **(D)** QoL.

Funnel plot analysis assessed publication bias (**Figure 8**). Depression outcomes showed symmetric distribution around the midline, indicating minimal bias risk (**Figure 8A**). Anxiety (**Figure 8B**), sleep (**Figure 8C**), and QoL (**Figure 8D**) showed asymmetric distributions, suggesting possible publication bias, likely due to the small number of studies.

Node-splitting analysis revealed local inconsistencies in anxiety and QoL outcomes (P < 0.05 for all), reflecting potential discrepancies between direct and indirect comparisons for certain interventions.

### 3.8 GRADE classification of quality of evidence

Evaluation of evidence quality using the CINeMA framework (**Supplementary Table S2**) revealed considerable limitations across the evidence base for exercise interventions targeting depression, anxiety, sleep, and QoL, with pervasive heterogeneity undermining confidence in findings. Depression outcomes demonstrated predominantly weak evidence quality. While SS versus control achieved moderate confidence—a notable exception—most comparisons received low to very low ratings. Heterogeneity and within-study bias consistently compromised evidence quality across interventions. The evidence supporting anxiety interventions proved particularly fragile. All comparisons yielded low or very low confidence ratings, primarily due to imprecision compounded by heterogeneity. This weakness in the evidence base highlights fundamental gaps in our understanding of exercise effects on anxiety symptoms. Sleep outcomes reflected similar limitations. Although MBE, ME, and SS versus control attained low confidence ratings, persistent reporting bias and heterogeneity substantially eroded the reliability of these findings. QoL evidence emerged somewhat more robust. Aerobic exercise versus control achieved moderate confidence, representing one of the stronger findings in this analysis. However, alternative interventions including dance and mind-body exercises remained constrained by low to very low confidence ratings, undermined by both within-study and reporting bias.

These findings illuminate a paradox: while exercise interventions show promise for improving mental health outcomes, the evidence supporting their use remains tentative, especially for indirect comparisons where uncertainty intensifies. Advancing this field requires methodologically rigorous studies with adequate power, transparent reporting, and systematic investigation of heterogeneity sources to establish a more definitive evidence base.

## 4 Discussion

This NMA evaluated the comparative effectiveness of exercise interventions on depression, anxiety, sleep quality, and QoL in cognitively impaired individuals. Based on 40 studies involving 2,937 participants, this is the first NMA to compare exercise effects across these four outcomes in this population. Results confirm beneficial effects of multiple exercise types on all four outcomes. Exergaming may be the most effective for depression and anxiety symptoms. For sleep quality, ME, MBE, and AE showed better benefits. MBE, ME, and dance were more effective for QoL.

**Figure 9:**
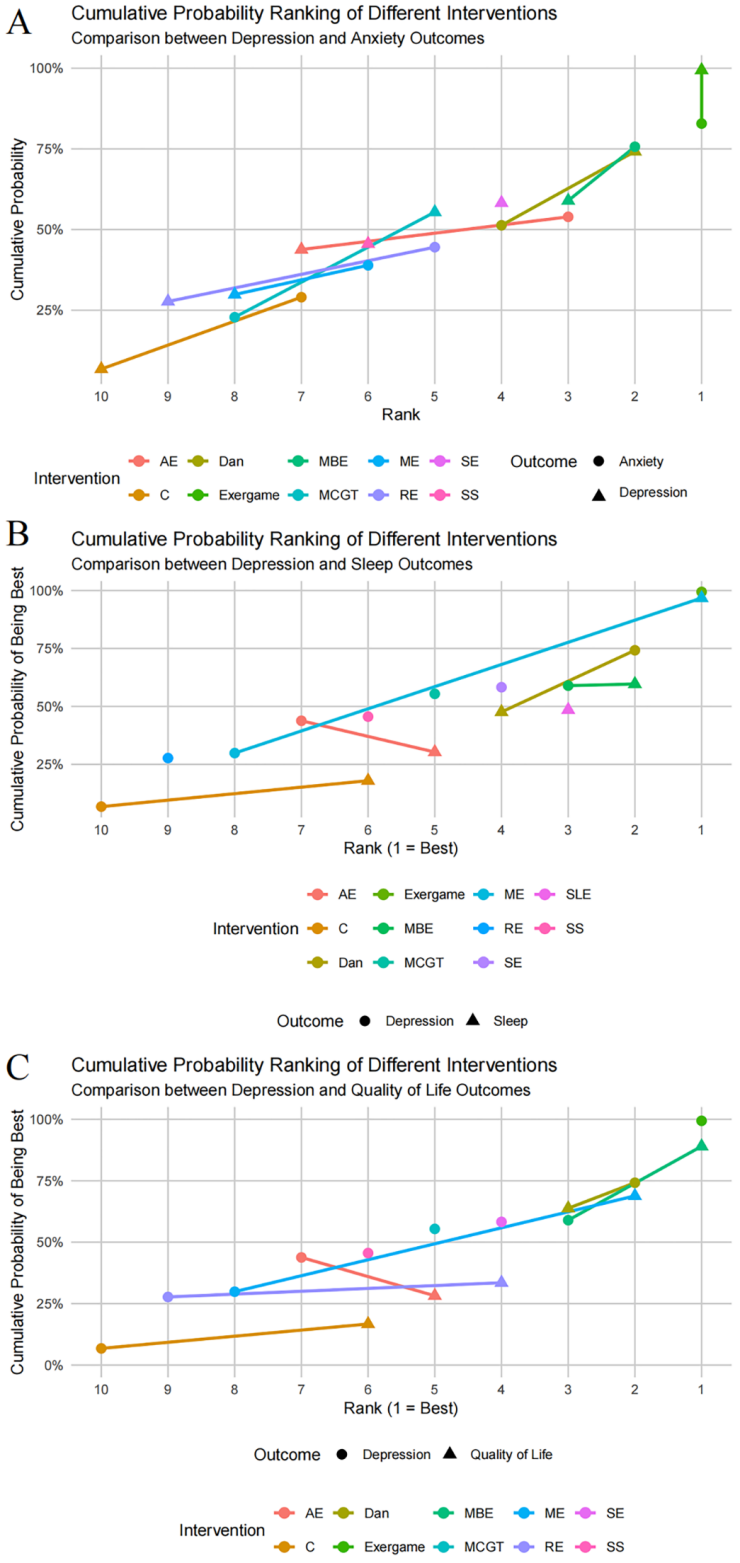
Cluster maps comparing optimal interventions: **(A)** Depression and anxiety; **(B)** Depression and sleep; **(C)** Depression and QoL.

Cumulative probability rankings identified optimal intervention strategies for combined outcomes. Exergaming demonstrated superior outcomes for both depression and anxiety, suggesting potential synergistic effects. In dementia patients, exergaming retained effectiveness, though its role in dementia remains uncertain due to limited data. Dance showed promising effects for both depression and sleep in MCI populations, while ME effectively improved both depression and QoL. Different exercise modalities may exert varying therapeutic mechanisms, contributing to their differential effectiveness across symptom domains (**Figure 9**). SUCRA values provide quantitative rankings but depend on consistency assumptions and require cautious interpretation. Significant local inconsistencies were observed in anxiety and quality of life networks, indicating discrepancies between direct and indirect evidence. Several factors may explain these inconsistencies. First, intervention protocols varied within categories; mind-body exercise studies included Tai Chi and Qigong with different intensities, frequencies, and psychological engagement levels. Second, risk of bias assessment revealed concerns regarding randomization, blinding, and selective reporting that may have systematically affected direct comparisons. Third, the relatively sparse networks for anxiety and quality of life, with limited studies and comparison links, increased uncertainty in indirect evidence and sensitivity to individual study effects. These limitations reduce confidence in SUCRA rankings for these outcomes.

The exergaming intervention employed the Dividat Senso system (Dividat, Schindellegi, Switzerland), featuring a 1.13 m × 1.13 m pressure-sensitive platform with strain gauges recording at 50 Hz. The system detected four-directional steps (left, right, forward, backward) with waist-height support bars for stability. Connected via USB to a computer and 43-inch display (LG 43LJ500V), participants interacted through stepping on directional arrows. Lateral movements required the corresponding limb, while forward/backward steps used the preferred limb. The system provided real-time visual, auditory, and tactile feedback through platform vibration.

Depression frequently accompanies cognitive impairment, with neurochemical changes affecting the hippocampus and anterior cingulate cortex contributing to both memory decline and this association (60). Exercise may mitigate these effects through multiple mechanisms including hormonal modulation, neuroplasticity enhancement, and physical function improvements (61). Different exercise types may activate distinct neurobiological pathways, explaining their varying effectiveness across symptom domains (62, 63). Previous meta-analyses demonstrated that physical activity alleviates depression in both AD and MCI populations, consistent with our findings (31, 64).

Both dance and AE can improve depression and mental state in elderly individuals with cognitive impairments. However, dance, as an economically effective multimodal intervention, is more effective in improving cognitive impairments and reducing depression and anxiety levels compared to single aerobic exercise (56, 65). Throughout the intervention process, music therapy accompanies exercise, with changes in music rhythm directly affecting exercise intensity. Music induces dopamine release, significantly improving emotional responses. When exercise is performed in sync with music rhythm, physical function is further enhanced. An inverted U-shaped relationship exists between music pitch segmentation and exercise scores, indicating that combining exercise with music therapy is more beneficial than single exercise. This combination effectively alleviates exercise fatigue and psychological depression and anxiety symptoms while improving cognitive dysfunction. Dance activities often accompany music rhythm, and listening to music is closely linked to neurochemical changes related to stress reduction. These changes include increased endogenous opioid substances and dopamine release, as well as decreased cortisol levels. Studies have observed increased cortical thickness in the temporal lobe region of dancers and musicians (66). Wang (67) found significant differences in left and right caudate gray matter volume in patients with mood disorders, with increased gray matter volume associated with positive emotions. Athletes show higher gray matter volume in somatosensory and visual spatial regions compared to non-athletes, attributed to microstructural changes in neuronal components and neuroglial cells (68). Individuals with smaller gray matter volume in the left hippocampus often experience more negative emotions and rumination. Dance intervention, through its unique comprehensive effects, can significantly reduce depression and anxiety levels. These results align with previous meta-analyses showing that dance interventions can improve depression in patients with cognitive impairment, but more randomized controlled trials are needed to validate these effects (69).

MBE emphasize the connection between mental focus and physical movement to enhance overall health and well-being (70). Tai Chi, as an aerobic MBE, significantly contributes to improved mental health. Research indicates that Tai Chi enhances cognitive performance in individuals with mild dementia and alleviates depression and behavioral symptoms (40). The practice requires participants to focus on breathing and body movements, helping reduce distractions, improve attention, and alleviate psychological stress and anxiety. The movements and breathing rhythm of Tai Chi promote relaxation and blood circulation, improving brain function and emotional regulation. The cultural and philosophical aspects of Tai Chi may also positively influence participants’ psychological state, helping them face life challenges with a more peaceful mindset. These findings align with previous studies (71). Yeh et al. (72)found that Tai Chi benefits psychological functions by improving mood and reducing anxiety and depression. Osypiuk et al. (73)indicated a bidirectional relationship between body posture and emotions, suggesting that Tai Chi’s slow, fluid movements help activate emotional regulation mechanisms. Multiple physiological and psychological mechanisms may explain exercise-related reductions in depressive symptoms, including increased self-efficacy, reduced stress responses, and beneficial effects on neurotransmitters, though exact mechanisms remain unclear.

Limited evidence exists regarding exercise effects on sleep disorders in patients with cognitive impairment.

However, studies suggest physical activity improves self-assessed sleep quality, reduces sleep onset time, and increases sleep duration, even at low intensities (74). Nascimento et al. (28)found that multimodal exercise programs effectively reduce sleep disorders and improve daily functioning in patients with cognitive impairment. Age-related sleep structure changes, particularly in individuals with neurodegenerative conditions, often contribute to psychological disorders that underlie sleep problems (75).

Sanghee et al. (24)demonstrated that Qigong exercise, as a mind-body practice, effectively alleviates non-motor symptoms in PD patients, particularly improving sleep quality through its breathing training component with potential as rehabilitative treatment. Research also shows that structured limb exercise programs impact cognitive function through mediating pathways involving depression symptoms, sleep quality, and processing speed, accounting for 69.22% of cognitive improvement variance (36). Sleep quality showed the strongest correlation with cognitive function, indicating that structured exercises can effectively maintain cognitive function and improve related symptoms in mild AD.

Higher physical activity levels correlate positively with better health-related QoL, while low activity levels typically result in poorer QoL. Regular physical activity contributes to enhanced physical, mental, and social functioning, which in turn facilitates better overall QoL (76). Therefore, active engagement in physical activity is crucial for preserving and improving QoL.Exercise improves QoL in patients with cognitive impairment by promoting neurogenesis and neurorestoration, enhancing BDNF expression, and increasing vagal tone (77, 78). It supports neuronal survival, increases resistance to brain damage, improves synaptic plasticity, strengthens functional connectivity across brain regions, and may correct structural brain abnormalities (79–84). Exercise also enhances mood, sleep quality, and comprehensively promotes cognitive performance, thereby alleviating cognitive decline symptoms and improving daily functioning and overall well-being. For patients with cognitive impairment, habitual exercise increases heart rate reserve and cerebral blood flow velocity, providing additional health benefits. Future research should incorporate pathway-based intervention strategies to maximize cognitive improvements.

Several limitations must be acknowledged. First, CINeMA assessment revealed low to very low quality evidence for most comparisons, with substantial heterogeneity arising from variations in intervention protocols, equipment, and study settings. While this heterogeneity limits internal consistency, it reflects real-world diversity and enhances external validity. Local inconsistencies in QoL outcomes likely resulted from the limited number of trials, necessitating additional RCTs. Second, although SMD enabled cross-scale pooling, their clinical meaningfulness remains unclear, limiting practical interpretation. Meta-regression failed to identify age, baseline status, or intervention duration as heterogeneity sources, suggesting unmeasured factors such as adherence or social context may contribute. Sparse network nodes compromised random-effects model stability, increasing result uncertainty. Third, absence of direct evidence for many intervention comparisons necessitated reliance on indirect estimates, potentially violating consistency assumptions and reducing credibility. Finally, restriction to English-language publications may have excluded relevant studies.

## 5 Conclusion

This NMA synthesizes current evidence on exercise interventions in patients with cognitive impairment. Exergaming, dance, and MBE effectively reduced depression and anxiety. ME and MBE improved sleep quality, while MBE, dance, and ME enhanced quality of life. Exergaming produced the strongest effect on depression and anxiety, dance was most effective for depression and sleep, and ME yielded the greatest improvement in depression and QoL.

Given the methodological limitations and sparse evidence base, these findings require cautious interpretation. Large-scale RCTs are needed to establish the relative efficacy of exercise modalities and inform evidence-based clinical guidelines.

## Data Availability

The original contributions presented in the study are included in the article/**Supplementary Material**. Further inquiries can be directed to the corresponding author.

## Supplementary material

SUPPLEMENTARY FILE S1 Search strategy for network meta-analysis. Characteristics of the included randomized controlled trials. GRADE classification of quality of evidence.
