# Supplementary material for: Impact of exercise intervention on depression, anxiety, sleep and quality of life in patients with cognitive impairment: a systematic review and network meta-analysis
Source: Front Psychiatry. 2025 Nov 17;16:1666157. doi: 10.3389/fpsyt.2025.1666157 (PMC12666691; doi:10.3389/fpsyt.2025.1666157)
Supplement: SUPPLEMENTARY FILE S1 — Search strategy for network meta-analysis. Characteristics of the included randomized controlled trials. GRADE classification of quality of evidence. [file SupplementaryFile1.docx]

Supplementary Material

Content

[1 File S1: Search strategy for network meta-analysis. 2](#_Toc211351270)

[1.1 Search strategy of Pubmed. 2](#_Toc211351271)

[1.2 Search strategy of Embase. 2](#_Toc211351272)

[1.3 Search strategy of Cochrane Library. 3](#_Toc211351273)

[1.4 Search strategy of Web of Science. 3](#_Toc211351274)

[2 Table S1 Characteristics of the included randomised controlled trials 5](#_Toc211351275)

[3 Table S2 GRADE classification of quality of evidence. 10](#_Toc211351276)

# File S1: Search strategy for network meta-analysis.

## Search strategy of Pubmed.

| No. | Search items |
| --- | --- |
| #1 | (Exercise OR Sports[MeSH Terms]) OR (Exercise*[Title/Abstract] OR Physical Activit*[Title/Abstract] OR Sport*[Title/Abstract] OR Athletic*[Title/Abstract] OR training[Title/Abstract]) |
| #2 | ((Cognitive Dysfunction OR Dementia[MeSH Terms]) OR (Cognitive Dysfunction*[Title/Abstract] OR Cognitive Disorder*[Title/Abstract] OR Cognitive Impairment*[Title/Abstract] OR Mild Cognitive Impairment*[Title/Abstract] OR Cognitive Decline*[Title/Abstract] OR Mental Deterioration*[Title/Abstract])) OR (Dementia*[Title/Abstract] OR Amentia*[Title/Abstract] OR Alzheimer∗[Title/Abstract]) |
| #3 | (((Sleep OR Anxiety OR Depression[MeSH Terms]) OR (Sleep*[Title/Abstract])) OR (Angst[Title/Abstract] OR Nervousness[Title/Abstract] OR Hypervigilance[Title/Abstract] OR Anxiet*[Title/Abstract] OR Anxiousness[Title/Abstract])) OR (Depress*[Title/Abstract]) |
| #4 | #1 AND #2 AND #3 |
| #5 | Random*[Title/Abstract] OR Clinic*[Title/Abstract] OR Trial*[Title/Abstract] OR Control[Title/Abstract] |
| #6 | #4 AND #5 |

## Search strategy of Embase.

| No. | Search items |
| --- | --- |
| #1 | 'exercise'/exp OR 'sport'/exp OR exercise*:ab,ti OR 'physical activit*':ab,ti OR sport*:ab,ti OR athletic*:ab,ti OR training:ab,ti |
| #2 | 'cognitive defect'/exp OR 'dementia'/exp OR 'cognitive dysfunction*':ab,ti OR 'cognitive disorder*':ab,ti OR 'cognitive impairment*':ab,ti OR 'mild cognitive impairment*':ab,ti OR 'cognitive decline*':ab,ti OR 'mental deterioration*':ab,ti OR dementia*:ab,ti OR amentia*:ab,ti OR alzheimer∗:ab,ti |
| #3 | 'sleep'/exp OR 'anxiety'/exp OR 'depression'/exp OR sleep*:ab,ti OR angst:ab,ti OR nervousness:ab,ti OR hypervigilance:ab,ti OR anxiet*:ab,ti OR anxiousness:ab,ti OR depress*:ab,ti |
| #4 | #1 AND #2 AND #3 |
| #5 | random*:ab,ti OR clinic*:ab,ti OR trial*:ab,ti OR control:ab,ti |
| #6 | #4 AND #5 |

## Search strategy of Cochrane Library.

| No. | Search items |
| --- | --- |
| #1 | MeSH descriptor: [Exercise] explode all trees |
| #2 | MeSH descriptor: [Sports] explode all trees |
| #3 | (Exercise* OR Physical Activit*):ti,ab,kw OR (Sport* OR Athletic* OR Training):ti,ab,kw |
| #4 | #1 OR #2 OR #3 |
| #5 | MeSH descriptor: [Cognitive Dysfunction] explode all trees |
| #6 | MeSH descriptor: [Dementia] explode all trees |
| #7 | (Dementia* OR Amentia* OR Alzheimer∗):ti,ab,kw OR (Cognitive Disorder* OR Cognitive Dysfunction* OR Cognitive Impairment* OR Mild Cognitive Impairment* OR Cognitive Decline* OR Mental Deterioration*):ti,ab,kw |
| #8 | #5 OR #6 OR #7 |
| #9 | MeSH descriptor: [Sleep] explode all trees |
| #10 | MeSH descriptor: [Anxiety] explode all trees |
| #11 | MeSH descriptor: [Depression] explode all trees |
| #12 | (Depress*):ti,ab,kw OR (Angst OR Nervousness OR Hypervigilance OR Anxiet* OR Anxiousness):ti,ab,kw OR (Sleep*):ti,ab,kw |
| #13 | #9 OR #10 OR #11 OR #12 |
| #14 | #4 AND #8 AND #13 |

## Search strategy of Web of Science.

| No. | Search items |
| --- | --- |
| #1 | TS=(Exercise* OR Physical Activit* OR Sport* OR Athletic* OR training) |
| #2 | (TS=(Cognitive Dysfunction* OR Cognitive Disorder* OR Cognitive Impairment* OR Mild Cognitive Impairment* OR Cognitive Decline* OR Mental Deterioration*)) OR TS=(Dementia* OR Amentia* OR Alzheimer∗) |
| #3 | ((TS=(Sleep*)) OR TS=(Angst OR Nervousness OR Hypervigilance OR Anxiet* OR Anxiousness)) OR TS=(Depress*) |
| #4 | #3 AND #2 AND #1 |
| #5 | TS=(Random* OR Clinic* OR Trial* OR Control ) |
| #6 | #5 AND #4 |

**2 Table S1 Characteristics of the included randomised controlled trials**

| Author | Year | Country | Age T | Age C | N T | N C | Disease | Diagnostic criteria | Treatment T | Treatment C | Outcome |
| --- | --- | --- | --- | --- | --- | --- | --- | --- | --- | --- | --- |
| Zhang | 2023 | China | 66.67 ± 6.04 | 69.75 ± 7.02(CG) | 14 | 14(CG) | MCI | MMSE, Simple memory test > 13 | ME | C | depression |
|  |  |  |  | 66.22 ± 5.51(WG) | 14(WG) |  |  |  | AE |  |  |
| Morris | 2017 | America | 71.4±8.4 | 74.4±6.7 | 34 | 34 | AD | CDR(0.5or1) | AE | C | depression |
| Abd El-Kader | 2016 | Saudi Arabia | 68.94 ± 5.76 | 69.13± 6.12 | 20 | 20 | AD |  | AE | C | depression, anxiety, quality of life |
| Moon | 2020 | America | 66.4±8.1 | 65.9±5.4 | 8 | 9 | PD | MMSE | MBE | C | sleep, depression |
| Akbuga Koc | 2024 | Turkey | 77.8±6.7 | | 20(PEG) 20 (PE+CSG) | 20 | AD | MMSE, MoCA | ME | C | depression, quality of life |
| Thunga | 2022 | India | 69±8.03 | 68.08±6.2 | 12 | 12 | PD |  | MBE | C | depression |
| Bardopoulou | 2024 | Greece | 81±6.8 | 80.1±7.2 | 19 | 19 | dementia | FRSSD, MMSE | SE | C | depression |
| Nascimento | 2014 | Brazil | PD 67.8±6.8 AD 76.8±6.8 | PD 66.3±8.1 AD 77.9±5.9 | PD 17 AD 14 | PD 17 AD 16 | PD/AD | DSM-IV-TR\CDR | ME | C | sleep |
| Song | 2024 | China | 76.71±5.96 | 75.20±6.63 | 45 | 44 | MCI | MoCA, PSQI | AE | C | sleep |
| Haghighi | 2023 | Iran | 69.92±8.16 | 72.92±6.17 | 12 | 13 | AD |  | ME | C | depression |
| Lok | 2023 | **Turkey** | 72.80±3.99 | 73.88±4.85 | 36 | 36 | AD | MMT | AE | C | depression |
| Zhu | 2020 | China | 68.53±1.90 | 67.77±1.72 | 19 | 22 | PD | MoCA | MBE | ME | sleep, depression, anxiety, quality of life |
| Adam | 2016 | **Malaysia** |  |  | 40 | 44 | cognitive impairment | MMSE | Dan | C | depression, anxiety, quality of life |
| Lin | 2022 | Taiwan, China | 84.09±5.60 | 83.00±7.20 | 44 | 40 | cognitive impairment | MMSE | AE | C | depression |
| Ben Ayed | 2024 | **Tunisia** | AG 70.04±0.86 ACT 69.32±0.90 | 71.04±0.90 | AT 27 ACT 25 | 26 | AD | MMSE | AE | C | depression, quality of life |
| Wang | 2020 | China | 68.37±5.27 | 68.24±5.15 | 57 | 54 | MCI | MMSE, MoCA | AE | C | depression, sleep |
| Sánchez-Sánchez | 2022 | **Spain** | 84.15 ± 4.76 | 83.99 ± 4.80 | 88 | 100 | MCI | MoCA | ME | C | depression |
| Boström | 2016 | **Sweden** | 84.4 ± 6.2 | 85.9 ± 7.8 | 93 | 93 | dementia | MMSE | SE | C | depression |
| Cancela | 2016 | **Spain** | 80.63 ± 8.32 | 82.90 ± 7.42 | 73 | 116 | dementia | MEC | AE | C | depression |
| Song | 2019 | China | 76.22±5.76 | 75.33±6.78 | 60 | 60 | MCI | MoCA | AE | C | depression, sleep, quality of life |
| Huang | 2019 | China | 81.9 ± 6.0 | 81.9 ± 6.1 | 40 | 40 | dementia | Clinical Dementia  Rating score <2 | MBE | C | depression |
| Yu | 2022 | Hongkong, China | M1 63.5±7.0  M2 63.5±5.7  V1 63.4±5.2  V3 63.3±5.1 | 63.7±4.7 | M1 7 M3 7 V1 8 V3 8 | 8 | MCI | Hong Kong version  of Montreal Cognitive Assessment | AE | C | depression, anxiety，sleep |
| Li | 2022 | China | 66.33 ± 10.89 | 69.17 ± 6.48 | 18 | 18 | PD | meeting the clinical  diagnostic criteria for idiopathic PD by the UK Parkinson Disease Association Brain Bank; diagnosed with PD at levels 1–3 | MBE | C | depression，anxiety |
| Henskens | 2018 | Netherlands | EX-ADL 86.95±7.21  ADL 86.05±5.86  EX 85.14±4.64 | 84.73±4.55 | EX-ADL 22  ADL 21  EX 22 | 22 | Dementia |  | AE | C | depression |
| Bademli | 2019 | **Turkey** | 72.24 ± 7.16 | 70.67 ± 8.34 | 30 | 30 | MCI | PSQI | ME | C | sleep |
| Cavalcante | 2022 | **Brazil** | 71±6  71±6 | 71±4 | REI 22 RT 23 | 22 | cognitive impairment | MoCA | RE  REI | C | depression |
| Yang | 2022 | China | MCI 70.5±5.4 AD 73.1±5.2 | MCI 72.0±8.1 AD 75.9±8.0 | MCI 11 AD 10 | MCI 9 AD 9 | MCI/AD | CDR(0.5or1) | SS | C | depression，sleep |
| Hoffmann | 2015 | **Denmark** | 69.8±7.4 | 71.3±7.3 | 107 | 93 | AD | MMSE | AE | C | depression，quality of life |
| Chen | 2017 | Taiwan, China | 80.7±8.0 | 81.6±6.7 | 65 | 62 | dementia | MMSE | RE | C | depression |
| Solla | 2019 | **Taiwan** | 67.8±5.9 | 67.1±6.3 | 10 | 10 | PD | DSM-5 | Dan | C | depression |
| Chan | 2016 | China | 78.4±7.1 | 82.2±6.7 | 27 | 25 | cognitive impairment | MMSE | MBE | C | sleep |
| Chang | 2021 | China | 76.56 ± 3.60 | 75.94 ± 3.61 | 62 | 47 | MCI | MoCA | Dan | C | depression |
| Langoni | 2019 | **Brazil** | 72.6 ± 7.8 | 71.9 ± 7.9 | 26 | 26 | MCI | Katz index15 (score ⩾5)  and Pfeffer et al.’s16 functional assessment (score ⩽2) | ME | C | depression |
| Son | 2018 | **Korea** |  |  | 33 | 30 | PD | stage 1 ~ 3 in the  Hoehn & Yahr Scale | ME | C | depression, anxiety, quality of life |
| Burt | 2020 |  | 67.5±8.8 | 61.9±8.0 | 15 | 15 | PD |  | MCGT | AE | depression, anxiety |
| Liu | 2020 | Taiwan, China | 84.68 ± 6.74 | 86.77 ± 6.99 | 31 | 30 | dementia | MMSE | SE | AE | depression |
| Ayari | 2023 | **France** | 79.8 ± 7.7 | 77.2 ± 5.3 | 11 | 12 | cognitive impairment | MMSE GRECO | Dan | AE | depression, anxiety |
| Parial | 2023 | Hongkong, China | 63.33±4.54 | 64.27±5.91 | 30 | 30 | MCI | MoCA | Dan | AE | depression |
| Swinnen | 2021 | Sweden | 84.7±5.6 | 85.3±6.5 | 23 | 22 | neurocognitive  disorder | MoCA | Exergame | C | depression, anxiety |
| TingTing Wu | 2025 | China | 70.12 ± 0.96 | 68.18 ± 0.98 | 34 | 34 | MCI | MoCA, MMSE | RE | C | depression, anxiety, quality of life |

AE, aerobic exercise; ME, Multicomponent Exercise; RE, Resistance Exercise; MBE, Mind-body Exercise; SE, Strength Exercise; Dan, Dance; SS, Sport Stacking; MCGT, Music-Contingent Gait Training, REI, RE with instability; C, control.

**3 Table S2 GRADE classification of quality of evidence.**

| Comparison | Number of studies | Within-study bias | Reporting bias | Indirectness | Imprecision | Heterogeneity | Incoherence | Confidence rating | Reason(s) for downgrading |
| --- | --- | --- | --- | --- | --- | --- | --- | --- | --- |
| Depression | | | | | | | | | |
| AE:C | 11 | No concerns | Low risk | Some concerns | No concerns | Some concerns | No concerns | Low | ["Indirectness","Heterogeneity"] |
| AE:Dan | 2 | Some concerns | Low risk | Some concerns | Major concerns | Some concerns | No concerns | Very low | ["Within-study bias","Indirectness","Heterogeneity"] |
| AE:MCGT | 1 | No concerns | Low risk | No concerns | Major concerns | No concerns | No concerns | Very low | ["Imprecision"] |
| AE:ME | 1 | Some concerns | Low risk | No concerns | Some concerns | Some concerns | No concerns | Very low | ["Within-study bias","Imprecision","Heterogeneity"] |
| AE:SE | 1 | Some concerns | Low risk | No concerns | Some concerns | Some concerns | No concerns | Very low | ["Within-study bias","Imprecision","Heterogeneity"] |
| C:Dan | 3 | Some concerns | Low risk | Major concerns | Major concerns | Some concerns | No concerns | Very low | ["Within-study bias","Indirectness","Heterogeneity"] |
| C:Exergame | 1 | No concerns | Low risk | No concerns | Some concerns | Some concerns | No concerns | Low | ["Imprecision","Heterogeneity"] |
| C:MBE | 4 | Some concerns | Low risk | No concerns | No concerns | Some concerns | No concerns | Low | ["Within-study bias","Heterogeneity"] |
| C:ME | 7 | Some concerns | Low risk | No concerns | No concerns | Some concerns | No concerns | Low | ["Within-study bias","Heterogeneity"] |
| C:RE | 3 | Some concerns | Low risk | No concerns | No concerns | Some concerns | No concerns | Low | ["Within-study bias","Heterogeneity"] |
| C:SE | 2 | Major concerns | Low risk | No concerns | No concerns | Some concerns | No concerns | Very low | ["Within-study bias","Heterogeneity"] |
| C:SS | 2 | No concerns | Low risk | No concerns | No concerns | Some concerns | No concerns | Moderate | ["Heterogeneity"] |
| MBE:ME | 1 | Some concerns | Low risk | No concerns | Some concerns | Some concerns | No concerns | Very low | ["Within-study bias","Imprecision","Heterogeneity"] |
| AE:Exergame | 0 | No concerns | Low risk | No concerns | Major concerns | No concerns | No concerns | Very low | ["Imprecision"] |
| AE:MBE | 0 | Some concerns | Low risk | No concerns | Major concerns | Some concerns | No concerns | Very low | ["Within-study bias","Imprecision","Heterogeneity"] |
| AE:RE | 0 | Some concerns | Low risk | No concerns | Major concerns | Some concerns | No concerns | Very low | ["Within-study bias","Imprecision","Heterogeneity"] |
| AE:SS | 0 | No concerns | Low risk | No concerns | Some concerns | No concerns | No concerns | Moderate | ["Imprecision"] |
| C:MCGT | 0 | No concerns | Low risk | No concerns | Some concerns | Some concerns | No concerns | Low | ["Imprecision","Heterogeneity"] |
| Dan:Exergame | 0 | No concerns | Low risk | Some concerns | Some concerns | No concerns | No concerns | Low | ["Indirectness","Imprecision"] |
| Dan:MBE | 0 | Some concerns | Low risk | Some concerns | Major concerns | Some concerns | No concerns | Very low | ["Within-study bias","Indirectness","Imprecision","Heterogeneity"] |
| Dan:MCGT | 0 | No concerns | Low risk | Some concerns | Some concerns | No concerns | No concerns | Low | ["Indirectness","Imprecision"] |
| Dan:ME | 0 | Some concerns | Low risk | Some concerns | No concerns | Some concerns | No concerns | Very low | ["Within-study bias","Indirectness","Heterogeneity"] |
| Dan:RE | 0 | Some concerns | Low risk | Some concerns | No concerns | Some concerns | No concerns | Very low | ["Within-study bias","Indirectness","Heterogeneity"] |
| Dan:SE | 0 | Some concerns | Low risk | Some concerns | Major concerns | Some concerns | No concerns | Very low | ["Within-study bias","Indirectness","Imprecision","Heterogeneity"] |
| Dan:SS | 0 | No concerns | Low risk | Some concerns | Major concerns | Some concerns | No concerns | Very low | ["Indirectness","Imprecision","Heterogeneity"] |
| Exergame:MBE | 0 | Some concerns | Low risk | No concerns | Major concerns | No concerns | No concerns | Very low | ["Within-study bias","Imprecision"] |
| Exergame:MCGT | 0 | No concerns | Low risk | No concerns | Major concerns | No concerns | No concerns | Very low | ["Imprecision"] |
| Exergame:ME | 0 | No concerns | Low risk | No concerns | Major concerns | No concerns | No concerns | Very low | ["Imprecision"] |
| Exergame:RE | 0 | No concerns | Low risk | No concerns | Major concerns | No concerns | No concerns | Very low | ["Imprecision"] |
| Exergame:SE | 0 | Some concerns | Low risk | No concerns | Major concerns | No concerns | No concerns | Very low | ["Within-study bias","Imprecision"] |
| Exergame:SS | 0 | No concerns | Low risk | No concerns | Major concerns | No concerns | No concerns | Very low | ["Imprecision"] |
| MBE:MCGT | 0 | Some concerns | Low risk | No concerns | Some concerns | No concerns | No concerns | Low | ["Within-study bias","Imprecision"] |
| MBE:RE | 0 | Some concerns | Low risk | No concerns | Some concerns | No concerns | No concerns | Low | ["Within-study bias","Imprecision"] |
| MBE:SE | 0 | Some concerns | Low risk | No concerns | Some concerns | No concerns | No concerns | Low | ["Within-study bias","Imprecision"] |
| MBE:SS | 0 | Some concerns | Low risk | No concerns | Some concerns | No concerns | No concerns | Low | ["Within-study bias","Imprecision"] |
| MCGT:ME | 0 | No concerns | Low risk | No concerns | Major concerns | No concerns | No concerns | Very low | ["Imprecision"] |
| MCGT:RE | 0 | No concerns | Low risk | No concerns | Major concerns | No concerns | No concerns | Very low | ["Imprecision"] |
| MCGT:SE | 0 | Some concerns | Low risk | No concerns | Major concerns | No concerns | No concerns | Very low | ["Within-study bias","Imprecision"] |
| MCGT:SS | 0 | No concerns | Low risk | No concerns | Major concerns | No concerns | No concerns | Very low | ["Imprecision"] |
| ME:RE | 0 | Some concerns | Low risk | No concerns | Some concerns | No concerns | No concerns | Low | ["Within-study bias","Imprecision"] |
| ME:SE | 0 | Some concerns | Low risk | No concerns | Major concerns | Some concerns | No concerns | Very low | ["Within-study bias","Imprecision","Heterogeneity"] |
| ME:SS | 0 | No concerns | Low risk | No concerns | Some concerns | No concerns | No concerns | Moderate | ["Imprecision"] |
| RE:SE | 0 | Some concerns | Low risk | No concerns | Some concerns | No concerns | No concerns | Low | ["Within-study bias","Imprecision"] |
| RE:SS | 0 | No concerns | Low risk | No concerns | Some concerns | No concerns | No concerns | Moderate | ["Imprecision"] |
| SE:SS | 0 | Some concerns | Low risk | No concerns | Some concerns | No concerns | No concerns | Low | ["Within-study bias","Imprecision"] |
| Anxiety | | | | | | | | | |
| AE:C | 2 | Some concerns | Some concerns | No concerns | No concerns | Some concerns | No concerns | Low | ["Within-study bias","Heterogeneity"] |
| AE:Dan | 1 | Some concerns | Some concerns | No concerns | No concerns | Major concerns | No concerns | Very low | ["Within-study bias","Heterogeneity"] |
| C:Dan | 1 | Major concerns | Some concerns | No concerns | Some concerns | Some concerns | No concerns | Very low | ["Within-study bias","Imprecision","Heterogeneity"] |
| C:Exergame | 1 | No concerns | Some concerns | No concerns | Some concerns | Some concerns | No concerns | Low | ["Imprecision","Heterogeneity"] |
| C:MBE | 2 | Some concerns | Some concerns | No concerns | Some concerns | Some concerns | No concerns | Very low | ["Within-study bias","Imprecision","Heterogeneity"] |
| C:MCGT | 1 | No concerns | Some concerns | No concerns | Some concerns | Some concerns | No concerns | Low | ["Imprecision","Heterogeneity"] |
| C:ME | 1 | Major concerns | Some concerns | No concerns | No concerns | Major concerns | No concerns | Very low | ["Within-study bias","Heterogeneity"] |
| C:RE | 1 | No concerns | Some concerns | No concerns | Some concerns | Some concerns | No concerns | Low | ["Imprecision","Heterogeneity"] |
| MBE:ME | 1 | Major concerns | Some concerns | No concerns | Some concerns | Some concerns | No concerns | Very low | ["Within-study bias","Imprecision","Heterogeneity"] |
| AE:Exergame | 0 | No concerns | Some concerns | No concerns | Major concerns | No concerns | No concerns | Very low | ["Imprecision"] |
| AE:MBE | 0 | Some concerns | Some concerns | No concerns | Some concerns | Some concerns | No concerns | Very low | ["Within-study bias","Imprecision","Heterogeneity"] |
| AE:MCGT | 0 | No concerns | Some concerns | No concerns | Some concerns | Some concerns | No concerns | Low | ["Imprecision","Heterogeneity"] |
| AE:ME | 0 | Some concerns | Some concerns | No concerns | Some concerns | Some concerns | No concerns | Very low | ["Within-study bias","Imprecision","Heterogeneity"] |
| AE:RE | 0 | No concerns | Some concerns | No concerns | Some concerns | Some concerns | No concerns | Low | ["Imprecision","Heterogeneity"] |
| Dan:Exergame | 0 | Some concerns | Some concerns | No concerns | Major concerns | No concerns | No concerns | Very low | ["Within-study bias","Imprecision"] |
| Dan:MBE | 0 | Some concerns | Some concerns | No concerns | Some concerns | Some concerns | No concerns | Very low | ["Within-study bias","Imprecision","Heterogeneity"] |
| Dan:MCGT | 0 | Some concerns | Some concerns | No concerns | Some concerns | Some concerns | No concerns | Very low | ["Within-study bias","Imprecision","Heterogeneity"] |
| Dan:ME | 0 | Major concerns | Some concerns | No concerns | Some concerns | Some concerns | No concerns | Very low | ["Within-study bias","Imprecision","Heterogeneity"] |
| Dan:RE | 0 | Some concerns | Some concerns | No concerns | Some concerns | Some concerns | No concerns | Very low | ["Imprecision","Heterogeneity"] |
| Exergame:MBE | 0 | Some concerns | Some concerns | No concerns | Major concerns | No concerns | No concerns | Very low | ["Imprecision"] |
| Exergame:MCGT | 0 | No concerns | Some concerns | No concerns | Some concerns | Some concerns | No concerns | Low | ["Imprecision","Heterogeneity"] |
| Exergame:ME | 0 | Some concerns | Some concerns | No concerns | Some concerns | Some concerns | No concerns | Very low | ["Imprecision","Heterogeneity"] |
| Exergame:RE | 0 | No concerns | Some concerns | No concerns | Major concerns | No concerns | No concerns | Very low | ["Imprecision"] |
| MBE:MCGT | 0 | Some concerns | Some concerns | No concerns | Some concerns | Some concerns | No concerns | Very low | ["Imprecision","Heterogeneity"] |
| MBE:RE | 0 | Some concerns | Some concerns | No concerns | Some concerns | Some concerns | No concerns | Very low | ["Imprecision","Heterogeneity"] |
| MCGT:ME | 0 | Some concerns | Some concerns | No concerns | Some concerns | Some concerns | No concerns | Very low | ["Imprecision","Heterogeneity"] |
| MCGT:RE | 0 | No concerns | Some concerns | No concerns | Some concerns | Some concerns | No concerns | Low | ["Imprecision","Heterogeneity"] |
| ME:RE | 0 | Some concerns | Some concerns | No concerns | Some concerns | Some concerns | No concerns | Very low | ["Imprecision","Heterogeneity"] |
| Sleep | | | | | | | | | |
| AE:C | 2 | No concerns | Some concerns | No concerns | No concerns | Major concerns | No concerns | Very low | ["Reporting bias","Heterogeneity"] |
| C:Dan | 1 | No concerns | Some concerns | No concerns | No concerns | Major concerns | No concerns | Very low | ["Reporting bias","Heterogeneity"] |
| C:MBE | 2 | Some concerns | Some concerns | No concerns | No concerns | Some concerns | No concerns | Low | ["Within-study bias","Reporting bias","Heterogeneity"] |
| C:ME | 3 | Some concerns | Some concerns | No concerns | No concerns | Some concerns | No concerns | Low | ["Within-study bias","Reporting bias","Heterogeneity"] |
| C:SLE | 1 | Some concerns | Some concerns | No concerns | No concerns | Major concerns | No concerns | Very low | ["Within-study bias","Reporting bias","Heterogeneity"] |
| C:SS | 2 | No concerns | Some concerns | No concerns | No concerns | Some concerns | No concerns | Low | ["Reporting bias","Heterogeneity"] |
| MBE:ME | 1 | Some concerns | Some concerns | No concerns | No concerns | Some concerns | No concerns | Very low | ["Within-study bias","Reporting bias","Heterogeneity"] |
| AE:Dan | 0 | No concerns | Some concerns | No concerns | Some concerns | Some concerns | No concerns | Low | ["Reporting bias","Imprecision","Heterogeneity"] |
| AE:MBE | 0 | Some concerns | Some concerns | No concerns | No concerns | Major concerns | No concerns | Very low | ["Within-study bias","Reporting bias","Heterogeneity"] |
| AE:ME | 0 | Some concerns | Some concerns | No concerns | Some concerns | No concerns | No concerns | Low | ["Within-study bias","Reporting bias","Imprecision"] |
| AE:SLE | 0 | Some concerns | Some concerns | No concerns | Some concerns | Some concerns | No concerns | Very low | ["Within-study bias","Reporting bias","Imprecision","Heterogeneity"] |
| AE:SS | 0 | No concerns | Some concerns | No concerns | No concerns | Major concerns | No concerns | Low | ["Reporting bias","Heterogeneity"] |
| Dan:MBE | 0 | Some concerns | Some concerns | No concerns | No concerns | Major concerns | No concerns | Low | ["Within-study bias","Reporting bias","Heterogeneity"] |
| Dan:ME | 0 | Some concerns | Some concerns | No concerns | Some concerns | Some concerns | No concerns | Very low | ["Within-study bias","Reporting bias","Imprecision","Heterogeneity"] |
| Dan:SLE | 0 | Some concerns | Some concerns | No concerns | Major concerns | No concerns | No concerns | Very low | ["Within-study bias","Reporting bias","Imprecision"] |
| Dan:SS | 0 | No concerns | Some concerns | No concerns | No concerns | Major concerns | No concerns | Very low | ["Reporting bias","Heterogeneity"] |
| MBE:SLE | 0 | Some concerns | Some concerns | No concerns | Some concerns | Some concerns | No concerns | Very low | ["Within-study bias","Reporting bias","Imprecision","Heterogeneity"] |
| MBE:SS | 0 | Some concerns | Some concerns | No concerns | No concerns | Major concerns | No concerns | Very low | ["Within-study bias","Reporting bias"] |
| ME:SLE | 0 | Some concerns | Some concerns | No concerns | Some concerns | Some concerns | No concerns | Very low | ["Within-study bias","Reporting bias","Imprecision","Heterogeneity"] |
| ME:SS | 0 | Some concerns | Some concerns | No concerns | Some concerns | No concerns | No concerns | Very low | ["Within-study bias","Reporting bias","Imprecision"] |
| SLE:SS | 0 | Some concerns | Some concerns | No concerns | No concerns | Major concerns | No concerns | Very low | ["Within-study bias","Reporting bias","Heterogeneity"] |
| QoL | | | | | | | | | |
| AE:C | 4 | No concerns | Some concerns | No concerns | No concerns | Some concerns | No concerns | Moderate | ["Reporting bias","Heterogeneity"] |
| C:Dan | 1 | Major concerns | Some concerns | No concerns | No concerns | Some concerns | No concerns | Very low | ["Within-study bias","Reporting bias","Heterogeneity"] |
| C:MBE | 1 | Major concerns | Some concerns | No concerns | No concerns | Some concerns | No concerns | Very low | ["Within-study bias","Reporting bias","Heterogeneity"] |
| C:ME | 1 | Some concerns | Some concerns | No concerns | Some concerns | No concerns | No concerns | Low | ["Within-study bias","Reporting bias","Imprecision"] |
| C:RE | 1 | No concerns | Some concerns | No concerns | Some concerns | Some concerns | No concerns | Low | ["Reporting bias","Imprecision","Heterogeneity"] |
| MBE:ME | 1 | Major concerns | Some concerns | No concerns | Some concerns | Some concerns | No concerns | Very low | ["Within-study bias","Reporting bias","Imprecision","Heterogeneity"] |
| AE:Dan | 0 | Some concerns | Some concerns | No concerns | No concerns | Some concerns | No concerns | Low | ["Within-study bias","Reporting bias","Heterogeneity"] |
| AE:MBE | 0 | Some concerns | Some concerns | No concerns | Some concerns | Some concerns | No concerns | Very low | ["Within-study bias","Reporting bias","Imprecision","Heterogeneity"] |
| AE:ME | 0 | Some concerns | Some concerns | No concerns | Some concerns | Some concerns | No concerns | Very low | ["Within-study bias","Reporting bias","Imprecision","Heterogeneity"] |
| AE:RE | 0 | No concerns | Some concerns | No concerns | No concerns | Major concerns | No concerns | Very low | ["Reporting bias","Heterogeneity"] |
| Dan:MBE | 0 | Major concerns | Some concerns | No concerns | Some concerns | Some concerns | No concerns | Very low | ["Within-study bias","Reporting bias","Imprecision","Heterogeneity"] |
| Dan:ME | 0 | Some concerns | Some concerns | No concerns | Some concerns | Some concerns | No concerns | Very low | ["Within-study bias","Reporting bias","Imprecision","Heterogeneity"] |
| Dan:RE | 0 | Some concerns | Some concerns | No concerns | Some concerns | Some concerns | No concerns | Very low | ["Within-study bias","Reporting bias","Imprecision","Heterogeneity"] |
| MBE:RE | 0 | Some concerns | Some concerns | No concerns | Some concerns | Some concerns | No concerns | Very low | ["Within-study bias","Reporting bias","Imprecision","Heterogeneity"] |
| ME:RE | 0 | No concerns | Some concerns | No concerns | Some concerns | Some concerns | No concerns | Low | ["Reporting bias","Imprecision","Heterogeneity"] |
